# Physicochemical and Nutritional Properties of Vegetable Oils from Brazil Diversity and Their Applications in the Food Industry

**DOI:** 10.3390/foods13101565

**Published:** 2024-05-17

**Authors:** Kamila Leal Correa, Fernanda Brito de Carvalho-Guimarães, Erika Silva Mourão, Hellen Caroline Oliveira Santos, Suellen Christtine da Costa Sanches, Maria Louze Nobre Lamarão, Rayanne Rocha Pereira, Wagner Luiz Ramos Barbosa, Roseane Maria Ribeiro-Costa, Attilio Converti, José Otávio Carréra Silva-Júnior

**Affiliations:** 1 Laboratory R&D Pharmaceutical and Cosmetic, Federal University of Pará, Rua Augusto Correa 01, Belém 66075110, PA, Brazil; kamila.correa@ics.ufpa.br (K.L.C.); fernandabc_@hotmail.com (F.B.d.C.-G.); erikamourao10@gmail.com (E.S.M.); 2 Laboratory of Nanotechnology Pharmaceutical, Federal University of Pará, Rua Augusto Correa 01, Belém 66075110, PA, Brazil; hellencosantos@gmail.com (H.C.O.S.); suellen.sanches@yahoo.com.br (S.C.d.C.S.); louzelamarao@gmail.com (M.L.N.L.); rmrc@ufpa.br (R.M.R.-C.); 3 Laboratory of Pharmacognosy, Institute of Public Health—(ISCO), Federal University of Western Pará (UFOPA), Santarém 68040255, PA, Brazil; rayanne.pereira@ufopa.edu.br; 4 Laboratory of Chromatography and Mass Spectrometry, Federal University of Pará, Rua Augusto Correa 01, Belém 66075110, PA, Brazil; zweigw@gmail.com; 5 Department of Civil, Chemical and Environmental Engineering, Pole of Chemical Engineering, Via Opera Pia 15, 16145 Genoa, Italy; converti@unige.it

**Keywords:** physicochemical properties, thermogravimetry, pequi oil, açaí oil, passion fruit oil, guava oil

## Abstract

In this study, the oils of açaí, passion fruit, pequi, and guava were submitted to physicochemical analysis to investigate their potential application in the food industry. Gas chromatography associated with mass spectroscopy showed that oleic and linoleic acids are mainly responsible for the nutritional quality of açaí, passion fruit, pequi, and guava oils, which exhibited 46.71%, 38.11%, 43.78%, and 35.69% of the former fatty acid, and 18.93%, 47.64%, 20.90%, and 44.72% of the latter, respectively. The atherogenicity index of the oils varied from 0.11 to 0.65, while the thrombogenicity index was 0.93 for açaí, 0.35 for guava, and 0.3 for passion fruit oils, but 1.39 for pequi oil, suggesting that the use of the first three oils may lead to a low incidence of coronary heart disease. Thermogravimetry showed that all tested oils were thermally stable above 180 °C; therefore, they can be considered resistant to cooking and frying temperatures. In general, the results of this study highlight possible applications of these oils in the food industry, either in natura or in typical food production processes.

## 1. Introduction

The Brazilian territory is composed of five large biomes where a great diversity of oil plant species can be found. Vegetable oils obtained from these species have a unique chemical composition, which gives them interesting biological, nutritional, and physical–chemical properties for application in various industrial sectors, in particular the food industry [1,2,3]. The interest in vegetable oils lies mainly in their high content of monounsaturated fatty acids (MUFA) and polyunsaturated fatty acids (PUFA). Epidemiological studies show the strong relationship between the PUFA and MUFA consumption on the decrease in the occurrence of diseases such as cancer, diabetes, and coronary heart disease. Unsaturated fatty acids perform vital organic functions in the human body, participate in cell metabolism as precursors of various regulatory lipids, maintenance of cell membrane structure, fluidity, signaling, and cell-to-cell interaction. Because of these effects, fatty acids influence human health, welfare, and disease risk [4,5,6].

MUFA (omega-9) and PUFA (omega-6, -3) have several benefits for human health. However, it is important to have the adequate intake of omega-3 and 6, because the excess of omega-6 acids in the diet prevents adequate absorption of omega-3 acids and blocks the conversion of ALA (α-linolenic acid) to DHA/EPA. Recommendations for the omega-6/omega-3 ratio in the diet, suggested in several countries, show a coverage range of 4 to 5:1 [5,7]. In addition to biological benefits, MUFA are very important for the physical stability of vegetable oils and products obtained from vegetable oils rich in MUFA. Pereira et al. [1] stated that vegetable oils rich in oleic acid are good candidates for frying foods, due to the thermal stability of oleic acid [5]. Besides the biological benefits, the MUFA are so important to the physical stability of the vegetable oils and the products obtained by vegetable oils rich in MUFA. Pereira et al. [1] stated that vegetable oils rich in oleic acid are good candidates for fry-ing foods, due to the thermal stability of oleic acid.

*Caryocar brasiliense* (pequi), *Euterpe oleraceae* (açaí), *Passiflora* sp. (passion fruit), and *Psidium guajava* (guava) are common oilseed species in the Brazilian territory, and present great nutritional significance and economic importance in their regions of origin. *Caryocar brasiliense* is a typical Cerrado species, a vegetation characteristic of the Brazilian Midwest, also appearing in areas of the north and northeast. *C. brasiliense* belongs to the Caryocaraceae family; this family contains plant species throughout Central and South America, and in Brazil, this fruit is its main representative. *C. brasiliense* is popularly known as pequi, a drupe with its kernels wrapped in a fleshy pulp and yellowish mesocarp, whose local population uses it for the production of traditional dishes. It is a fruit rich in phenolic compounds, carotenoids, vitamins, and mono- and polyunsaturated fatty acids. The fruits of *C. brasiliense* are consumed after cooking, and the oil extracted from the fruits is used by the local population for cooking and frying [8,9,10].

*E. oleraceae*, belonging to the Arecaceae family, is a typical fruit of the Amazon region, where it is known as açaí. The açaí is a globose drupe with a purple or green epicarp; depending on the maturation, it has a pulpy mesocarp and a rounded epicarp that forms the fruit. In its region of origin, it is widely consumed in the form of juices, pulps, sweets, and ice creams, and plays an essential role in feeding the local population. It is a fruit rich in phenolic acids, flavonoids, essential fatty acids, and other nutrients and is considered a “superfruit” because of its high nutritional value. From the pulp the fixed açaí oil is extracted, which represents 50% of the total dry matter of the pulp. Açaí oil presents itself as a valuable product given its sensory properties and its potential health benefits, and it has been used by the cosmetic industry to produce soaps, shampoos, and moisturizers [11,12,13,14].

*Passiflora* sp. has 525 species, only 25 of which do not appear in the Americas. Brazil and Colombia have the greatest diversity of wild and commercial species of *Passiflora* sp.; usually, the fruits of these species receive the popular name of passion fruit. The root and aerial parts of passion fruit are used in many countries as anxiolytic, sedative, diuretic, and many other biological effects. The oil extracted from its seeds has great skin hydration power and is therefore widely used in the cosmetic industry. The pulp is rich in minerals such as iron, zinc, magnesium, phosphorus, and essential fatty acids, and bioactive compounds such as flavonoids, phenolic acids, and protocyanidins, which give it antioxidant activity [15,16,17,18].

Finally, the *Psidium guajava*, from the Myrtaceae family, popularly known as guava, is widely cultivated in Asia, Africa, and Central and South America. It is a fruit of great commercial importance because of its aroma and taste, being consumed *in natura* or processed in the form of juice, sweets, and ice cream. It is a nutritionally important fruit, a source of vitamin C, niacin, riboflavin, vitamin A, β-carotene, and lycopene. The seeds, by dry weight, contain 14% oil, 15% protein, and 13% amide, compounds, and flavonoids, including quercetin-3-O-β-D-(2″-O-galloyl-glucoside)-4′-O-vinylpropionate [19,20,21].

The use of these species as a source of mono- and polyunsaturated fatty acids is subject to physical and chemical characterization studies of the oils extracted from them. Techniques such as thermogravimetry, gas chromatography, and Rancimat, among others, allow researchers to obtain information on thermal stability of oils, fatty acid profile, and oxidative stability, respectively [2,22,23]. Therefore, in this research, we propose to study the physical and chemical characteristics of oils obtained from the species of *C. brasilienses*, *E. oleracea*, *Passiflora* sp., and *P. guava*, to study the potentiality of these oils for use in the food industry, whether in natura or processed.

## 2. Materials and Methods

### 2.1. Materials

The oils were extracted by the mechanical method of cold pressing at room temperature, where the pulps of the açaí and pequi fruits, as well as the guava and passion fruit seeds, were crushed without applying heat, meaning that their nutrients suffered minimal damage oxidation and loss due to heating and friction. Furthermore, the oil extraction was performed with the use of oil press (LDS R 135/2008, São Paulo, Brazil, 240 kg h^−1^). Three samples from different lots of crude açaí, guava, passion fruit, and pequi oil without addition of any preservative were provided by Amazon Oil Industry (Ananindeua, Pará, Brazil—https://amazonoil.com.br/), accessed on 30 January 2024.

### 2.2. Fatty Acid and Triacylglycerol Compositions

Fatty acid profile of samples was obtained by gas chromatography coupled to mass spectrometer (Gas chromatograph mass spectrometer ultra, CGMS-QP2010, Shimadzu, Japan). Conversion of triglycerides to methyl esters was performed via saponification and esterification with potassium hydroxide in methanol (0.1 M) and hydrochloric acid in methanol (0.12 M) [24]. Fatty acid profile was conducted according to the following specifications: column used SH-Rtx-5 30 m × 0.25 mm; entrainment gas: helium with flow of 1.5 mL/min; injection volume of 1 μL (split at 1:50 ratio); temperature gradient used was a heating ramp of 2 min at 60 °C; heating to 200 °C at the rate of 10 °C/min; heating to 240 °C at the rate of 2 °C/min and kept at that temperature for 24 min. Detector operated at temperature of 250 °C. Fatty acid peaks were identified from NIST 2008 mass spectral library database software (Shimadzu Inc., Kyoto, Japan) of QP2010 ULTRA mass detector. The fatty acid profile was performed in triplicate for each sample.

The fatty acid composition was used to predict the groups of TAGs in the non-interesterified sample with PrÓleos software 1.0 (Goias, Brazil), which uses a mathematical algorithm that describes the distribution of fatty acids in TAG molecules (Anon., 2015 https://projetos.extras.ufg.br/plames/#main, accessed on 25 February 2019) [25]. For the prediction, the average values of fatty acids with more than 1% of the total composition were used, and TAGs at predicted levels below 0.5% of the total were excluded. The composition of TAGs present in interesterified lipids was analyzed according to the 1,3-random, 2-random theory (non-random redistribution), and the 1,2,3-random theory (random redistribution), based on the analysis of region-specific distribution [1,25].

### 2.3. Atherogenicity and Thrombogenicity Indexes

FA compositions were used to evaluate the lipids’ nutritional quality through the atherogenicity (AI) and thrombogenicity indexes (TI) [26,27] defined in Equations (1) and (2), respectively:

(1) $$AI=\frac{C12:0+4\times C14:0+C16:0}{\sum MUFA+\sum FA\omega 6+\sum \omega 3}$$

(2) $$TI=\frac{C14:0+C16:0+C18:0}{0.5\times \sum MUFA+0.5\times \sum FA\omega 6+3\times \sum FA\omega 3}$$

where C12:0, C14:0, C16:0, and C18:0 are the relative percentage masses of lauric, myristic, palmitic, and stearic acids, respectively, MUFA is the relative percentage mass of monounsaturated fatty acids, and FAω6 and FAω3 are the relative percentage masses of omega-3 and omega-6 fatty acids, respectively.

These indexes indirectly indicate the ability of a substance to prevent the appearance of micro- and macro-coronary diseases and the tendency to form clots in the blood vessels, respectively. Particularly, the lower the AI and TI values, the better the food from the nutritional viewpoint of the lipid fraction.

### 2.4. Oil Quality Parameters

The physicochemical characterization of the oils was performed according to the official methods of the American Oil Chemists’ Society [28]. The acid, peroxide, and refractive indexes were evaluated according to the AOCS Cd3d-63, Cd 8b156 90, and Cc7-25 protocols, respectively, while the saponification and iodine indexes were determined by the recommended practices Cd 1c-85 and Cd 3a-94, respectively. The oxidative stability index (OSI) was evaluated by the Rancimat 743 (Metrohm, Herisau, Switzerland) equipment at 110 °C, under an air flow of 10 L/h using 5 g of oil, following the AOCS method Cd 12b-92 [28]. Oil viscosity was measured in a rotational viscometer (model Ct52, Schott-Gerate GmbH, Mainz, Germany). The kinematic viscosity was measured at a temperature of 40 °C [1]. Experimental analyses were performed in triplicate, and the results presented as means ± standard deviations. All the analyses were performed in triplicate for each sample.

### 2.5. FTIR-ATR

Absorption spectroscopy in the infrared region of samples was performed in a Fourier transform infrared (FTIR) spectrophotometer (IRPrestige-21, Shimadzu, Kyoto, Japan) with attenuated total reflection (ATR) coupled accessory, in the spectral region from 4000 to 600 cm^−1^ with a resolution of 4 cm^−1^ and 32 scans [12].

### 2.6. Thermogravimetry

Thermogravimetry (TG) was performed on a TGA analyzer (model 50/50H, Shimadzu, Kyoto, Japan). Briefly, 5 to 10 mg of the sample was weighed in a platinum crucible and heated from room temperature to 600 °C at a heating rate of 10 °C/min. The analyses were performed in synthetic air and nitrogen atmosphere [23].

## 3. Results and Discussion

### 3.1. Fatty Acid Composition, Nutritional Quality Indexes and Triacylglycerol Profile

The fatty acids (FAs) in vegetable oils are classified as saturated or unsaturated fatty acids. Gas chromatography associated with mass spectroscopy (GC/MS) made it possible to separate, quantify, and identify FAs present in açaí, guava, passion fruit, and pequi oils (Table 1). Açaí and pequi oils were mostly composed of oleic acid (46.72% and 38.80%, respectively), which was also found in high amounts (>37%) in the other oils (Table 1). On the other hand, passion fruit and guava oils were mostly composed of linoleic acid (47.63% and 44.72%, respectively), whose content in açaí and pequi oils was 18.83% and 19.39%, respectively (Table 1).

Vegetable oils rich in oleic acid are important for the food industry since their thermal stability allows their use in frying and cooking food. In addition, high contents of oleic and linoleic acids increase the shelf life and the nutritional value of foods. All the oil samples considered in this study had a high oleic acid content, mainly the pequi and açaí ones, in which it was (38.80% and 46.72%, respectively) higher than in 15–20% of grape seed oil and sea buckthorn oil (15–20%), but lower than in olive oil (55–80%) [29]. Olive oil is recommended for human consumption because it promotes health benefits, mainly for the cardiovascular system, which are attributed precisely to oleic acid. Furthermore, it is important to highlight that açaí, guava, passion fruit, and pequi oils are rich in monounsaturated fatty acids, such as oleic acid, which can help reduce cholesterol levels and even improve protection against cardiovascular diseases [29,30,31].

**Figure 1:**
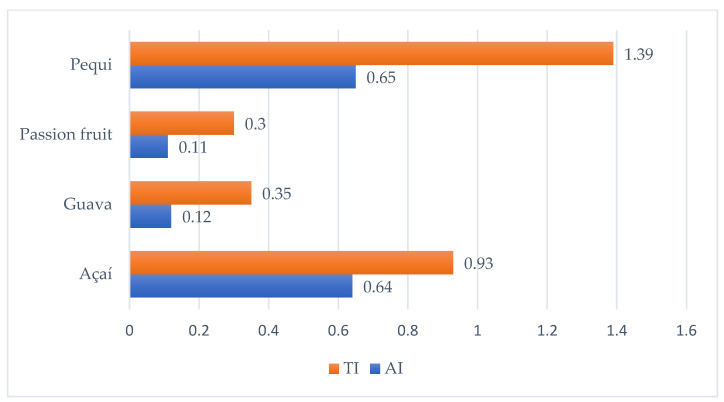
Atherogenicity and thrombogenicity indices of vegetable oils.

The use of vegetable oils in food preparation depends on their nutritional characteristics, which are directly influenced by their fatty acid profile. The atherogenicity (AI) and thrombogenicity (TI) indices proposed by [27] express the nutritional quality of vegetable oils and fats. Table 1 and Figure 1 show that the AI ranged from 0.11 to 0.65 and the TI ranged from 0.3 to 1.39. It is known that there are no AI and IT reference values for açaí, guava, passion fruit, and pequi oils; in this study, it was observed that the values found for passion fruit oil (AI: 011; IT: 0.3) and for guava oil (AI:0.12; IT: 0.35) are close to the AI and TI values reported for traditional cooking oils, such as olive oil (AI: 0.12; TI: 0.32), soybean (AI: 0.14; TI: 0.25), corn oils (AI: 0.17; TI: 0.37), sunflower (AI: 0.10; TI: 0.27), and cotton (AI: 0.30; TI: 0.58), which are lower due to the low concentration of SFA [32,33]. However, the values found for pequi oil (AI: 0.65; TI: 1.39) and açaí oil (AI: 0.64; TI: 0.93) are above the values reported for traditional cooking oils, but are in line with other studies on vegetable oils and tropical oils that have a greater amount of saturated fatty acids—such as palmitic acid—which guarantees greater stability of these oils, which are more resistant to oxidation at high temperatures, which increases its shelf life and contributes to its use in the food industry. Furthermore, these oils have levels that suggest a lower risk of causing cardiovascular diseases due to the high amount of MUFA [32,33].

The profile of triacylglycerols (TAGs) in the oil samples under study was determined by the method proposed by [25]. Table 2 shows that the most abundant TAGs were those composed of oleic and linoleic acids, regardless of the type of oil. TAG composition in açaí oil has already been described by Silva et al. [34], who found mainly triolein (OOO) followed by linoleo-diolein (OLO) and palmito-diolein (POO). The TAG profile detected in the present study for passion fruit oil, with lower content of trilinolein (LLL) than of dioleolinolein (OLL), is opposite to the one reported by [35] using mass spectroscopy. This difference may be related to intrinsic factors of the oil, such as the fruit maturation stage and extraction method. Finally, [36], who determined the TAG profile of the pequi oil, found that dioleopalmitin (POO) was its main constituent, which is consistent with the highest palmito-linoleo-stearin (PLS)/POO pair content detected in this study (Table 2).

### 3.2. Oily Quality Parameters

The quality parameters of tested oils are listed in Table 3. Among them, the acidity and peroxide indexes allow an assessment of the oxidation status of vegetable oils. Oils of açaí and pequi had acidity index values (6.17 and 5.25 mgKOH/g, respectively) higher than the maximum one (4 mg KOH/g) recommended by [37] for high-quality crude oils, while those of guava and passion fruit oils were well below the quality limit. The hot and humid climate of the Amazon region may have been responsible for the high acidity index of the açaí oil [12], while that of pequi oil, as suggested by Ribeiro et al. [38], can be ascribed to its extraction by mechanical press. On the other hand, all oils showed values of the peroxide index below the maximum threshold value (15 meq/kg) reported by the Codex Alimentarius for high-quality oils (Table 3).

The saponification, refractive, and iodine indexes allow us to identify the possible changes in sample. The saponification index ranged from 181.26 mg KOH/g for passion fruit oil to 191.01 mg KOH/g for guava oil. As observed by Pereira et al. [39], most of the oils exhibit values of this parameter in the range of 180–200 mg KOH/g, which is indicative of the presence of high molecular weight fatty acids in their composition.

As shown in Table 3, the refractive index, which grows with the increase in the length of the fatty acid chain and/or the decrease in the degree of unsaturation, ranged from 1.463 to 1.465, while the iodine index, which is directly proportional to the degree of unsaturation, was high for all the tested oils, ranging from 110.02 in the pequi oil to 134.25 in the guava one.

The oxidative stability index, also called oxidative induction period (OSI), indicates the development of lipid oxidative products and can be associated with oil deterioration during storage and exposure to heating [39,40]; it ranged from 10.05 ± 0.04 h for the passion fruit oil to 16.04 ± 0.01 h for the guava oil, with values around 15.49 ± 0.02 for the pequi and 15.24 ± 0.01 h for the açaí. All these values are higher than those reported for sunflower oil (6.21 h) [41] and within the range of those reported for almond oil (10.2–24.2) [42]. Finally, the lowest and highest kinematic viscosities were detected in the passion fruit oil (32.8 mm^2^/s) and açaí oil (43.0 mm^2^/s), consistent with their highest contents of unsaturated fatty acids (MUFA + PUFA = 85.88%) and saturated fatty acids (32.79%), respectively (Table 1).

### 3.3. FTIR-ATR

**Figure 2:**
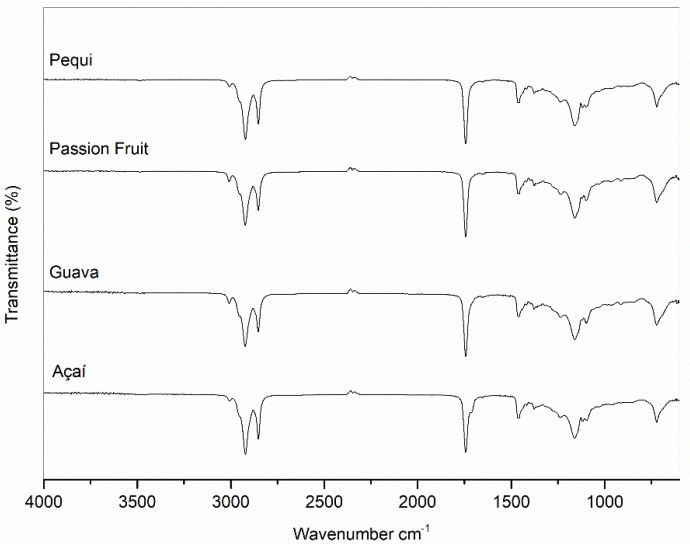
FTIR-ATR spectra of passion fruit, guava, açaí, and pequi oils with a resolution of 2 cm^−1^ and within the wavenumber range from 4000 to 600 cm^−1^.

The FTIR-ATR spectra of all the tested oils illustrated in Figure 2 show the prevalence of vibrational absorption bands in the region from 2850 to 3000 cm^−1^, corresponding to the C-H bond stretching, while the intense absorption band in the region from 1735 to 1750 cm^−1^ refers to the elongation of the carbonyl group (C=O). The absorption ranges close to 1465 and 1375 cm^−1^ show bands of the symmetrical angular deformations of the C-H bond of methylene (-CH_2_) [43] and terminal CH_3_ group, respectively, while the strong absorption band close to 1170 cm^−1^ can be ascribed to the stretching of the C-O-C bond. Finally, the medium-intensity absorption at 720 cm^−1^ is related to the asymmetric deformation of the C-H bond of methylene [12]. All these bands, which have been constantly observed in vegetable oils, come from the triglyceride molecules. FTIR is useful to observe the functional groups present in the sample. This observation is made by carefully analyzing the characteristic absorption bands of each functional group. As oils are mainly made up of triglycerides, different oils will present similar FTIR spectra, as the functional groups are always the same [43,44,45].

### 3.4. Thermal Analysis

**Figure 3:**
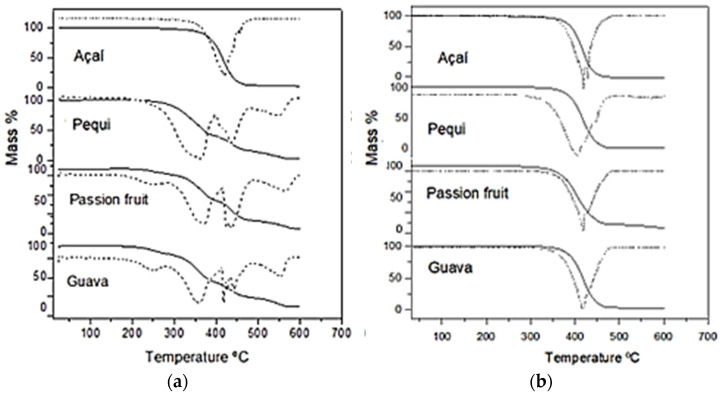
Thermogravimetry (TG) (continuous lines) and differential thermogravimetry (DTG) (dotted lines) curves of pequi, açaí, guava, and passion fruit oils in (**a**) synthetic air atmosphere, (**b**) nitrogen atmosphere.

The thermal behaviors of the studied oils can be seen in Figure 3.

In the synthetic air atmosphere, the initial decomposition temperatures of guava and passion fruit oils were 222.33 and 218.99 °C, while in the nitrogen atmosphere, they were 281.15 and 311.8 °C, respectively; on the other hand, decomposition of pequi and açaí oils started at temperatures as high as 298.30 and 382.60 °C in the synthetic air atmosphere, and even 343.60 and 332.32 °C in the nitrogen atmosphere, respectively (Table 4). These results indicate that, regardless of the atmosphere they were subjected to, the oils of passion fruit and guava exhibited lower thermal stability than the others.

Events that occur under a nitrogen atmosphere are usually due to the pyrolysis of triglycerides, and their rates are lower than in an oxidizing environment [46]. Although the thermogravimetry (TG) curves showed that all oils, when subjected to the nitrogen atmosphere, exhibited only one mass loss event, the differential thermogravimetric (DTG) ones revealed the occurrence of three simultaneous events, with the exception of açaí oils, for which the appearance of a tortuous peak did not allow them to establish their exact number (Figure 2).

The TG/DTG curves of the oil samples under the synthetic air atmosphere revealed the occurrence of two to four events. Pardauil et al. [46] attributed the first loss of mass (up to 290 °C) to the removal of volatile substances, moisture, and short-chain 12–16 carbon fatty acids. The first mass loss event may still be related to the smoke point of the oils under study, i.e., the release of volatile and low molecular weight compounds and free fatty acids. In frying processes with vegetable oils, for example, smoke is clearly seen emerging [47].

According to [48], events related to thermal decomposition of vegetable oils in an oxidizing atmosphere usually correspond to the degradation of polyunsaturated (200 to 380 °C), monounsaturated (380 to 480 °C), and saturated (480 to 600 °C) fatty acids. However, since there are other substances in addition to fatty acids, such as pigments, chlorophylls, carotenoids, terpenoids, vitamins, proteins, glycerides and non-glycerides, polyphenols, and secondary metabolites, among others, it is sometimes difficult to interpret the results of their thermogravimetry analysis [46]. An example of the high complexity of thermogravimetry of vegetable oils is given by açaí oil, which apparently exhibited, as previously mentioned, only one mass loss event in the TG analysis and a tortuous peak in the DTG one, likely caused by several simultaneous events.

The thermogravimetric study of vegetable oils is of great importance when they are intended for food application. Vegetable oils are used in various ways in the food industry, i.e., in their in natura form, emulsified, or even as media for cooking or frying [49,50]. Deep-fat frying is one of the most-used frying methods in food processing for its ease, agility of use, and low cost. Palm oil stands out for use in frying mainly because it starts to lose mass above 200 °C, which makes it possible to use in the typical frying temperature range (150–190 °C) [49,50]. All the oils studied suffered a mass loss greater than 10% at temperatures above 300 °C, although those of guava and passion fruit showed around 8 to 10% of mass loss between 200 and 270 °C (Table 4), likely due to volatilization of minor constituents such as short-chain fatty acids.

In the frying process, there is a mass transfer from the oil to the food. Therefore, the resulting food will be rich in the main constituents of the oil. For instance, foods fried with palm oil are usually rich in palmitic acid [49,50]. Oleic acid is a monounsaturated fatty acid whose health benefits are widely recognized and desired in foods. All the oils evaluated in this study had high content of oleic acid, which decreased in the following order: açaí (46.72%) > pequi (38.80%) > passion fruit (38.11%) > guava (37.90%) oil (Table 1). In addition, they also had promising contents of linoleic acid, which decreased in the following order: passion fruit (47.63%) > guava (44.72%) > pequi (19.39%) > açaí (18.83%) oil. However, this acid, despite its great health benefits, when subjected to elevated temperatures becomes more reactive and susceptible to oxidation, reducing the shelf life of fried food. Therefore, making a balance of the advantages and disadvantages of the tested oils when subjected to elevated temperatures, açaí oil stood out due not only to its thermal stability, but also to its favorable fatty acid composition.

## 4. Conclusions

The oils of açaí, pequi, guava, and passion fruit have in their composition the oleic and linoleic acids as the main fatty acids. The high content of oleic acid and linoleic acids is responsible for the high nutritional quality of these oils, evidenced by their low values of thrombogenicity and atherogenicity index. On the other hand, this composition makes these oils interesting for the production of food-grade nanoemulsions and lipid nanoparticles. Mixtures of these oils with solid lipids are an alternative to the oil phase of nanostructured lipid carriers rich in mono- and polyunsaturated fatty acids.

Regarding the physical and chemical characteristics, the oils showed values of acidity and peroxide indexes within what is recommended by the official regulations, obviously respecting their individual peculiarities. The other physicochemical characteristics, such as the acidity, saponification, refraction, and iodine indexes, confirmed the good quality of the oils for use in food preparation.

The results of the Fourier transform infrared spectroscopy showed typical bands of carboxylic acids and esters, depending on the nature of oil triacylglycerols. Finally, thermogravimetry studies revealed that oils, even if crude, have high thermal stability (all above 180 °C), which makes them suitable for frying and/or cooking food. The thermal stability (above 100 °C) of the oils opens up alternative applications as inputs for food products that require heating during production, such as gums/jujubes.

In general, the results of the present study showed that pequi, açaí, passion fruit, and guava oils have the potential to be used in gastronomic recipes outside their regions of origin and may be suitable for developing new fatty products in the food industry.

## Figures and Tables

**Table 1:** Fatty acid compositions (%), atherogenicity (AI), and thrombogenicity (TI) indexes of açaí, guava, passion fruit, and pequi oils.

Fatty Acid	Açaí	Guava	Passion Fruit	Pequi
Caprylic acid (C 8:0)	0.06 ± 0.01	0.03 ± 0.01	-	-
Capric acid (C 10:0)	0.03 ± 0.00	-	-	-
Lauric acid (C 12:0)	1.45 ± 0.02	-	-	-
Myristic acid (C 14:0)	5.70 ± 0.05	0.05 ± 0.02	-	0.16 ± 0.04
Palmitic acid (C 16:0)	18.52 ± 0.12	9.93 ± 0.01	9.23 ± 0.02	37.59 ± 0.01
Palmitoleic acid (C 16:1)	1.53 ± 0.03	0.07 ± 0.04	-	0.68 ± 0.01
Stearic acid (C 18:0)	7.05 ± 0.01	4.84 ± 0.02	3.68 ± 0.04	3.08 ± 0.02
Oleic acid (C 18:1)	46.72 ± 0.02	37.90 ± 0.03	38.11 ± 0.31	38.80 ± 0.03
Linoleic acid (C18:2)	18.83 ± 0.03	44.72 ± 0.05	47.63 ± 0.04	19.39 ± 0.02
Linolenic acid (C18:3)	-	0.19 ± 0.00	0.14 ± 0.01	-
Behenic acid (C22:0)	-	0.29 ± 0.04	-	-
SFA	32.81 ± 0.20	15.14 ± 0.01	12.91 ± 0.08	40.83 ± 0.08
MUFA	48.25 ± 0.05	37.97 ± 0.01	38.11 ± 0.44	39.48 ± 0.04
PUFA	18.83 ± 0.03	44.91 ± 0.05	47.77 ± 0.06	19.39 ± 0.02
AI	0.65	0.12	0.11	0.65
TI	0.93	0.35	0.30	1.39

Note: SFA = saturated fatty acids; MUFA = monounsaturated fatty acids; PUFA = polyunsaturated fatty acids; AI = atherogenicity index; TI = thrombogenicity index.

**Table 2:** Triacylglycerol composition of açaí, guava, passion fruit, and pequi oils.

Identifier/Shorthand	Açaí	Guava	Passion Fruit	Pequi
LLL/54:6	-	10.201	11.424	0.942
MOO/50:2	5.350	-	-	-
OLL/54:5	7.179	25.716	27.191	5.896
OLO/54:4	17.740	21.608	-	-
OOO/54:3	14.611	6.052	-	-
PLL/52:4	-	6.630	6.585	4.254
PLO/52:3	-	11.142	10.449	17.743
PLP/50:2	-	1.436	1.265	6.401
PLS/52:2	-	1.422	-	-
PLS-POO/52:2	-	-	5.152	19.760
POO/52:2	17.270	4.681	-	-
POP/50:1	6.805	-	1.004	13.350
POS/52:1	-	-	-	2.619
PPP/48:0	-	-	-	3.210
PSP/50:0	-	-	-	0.945
SLL/54:4	-	3.281	-	-
SLL-OLO/54:4	-	-	24.193	12.714
SLO/54:3	5.318	5.515	-	-
SLO-OOO/54:3	-	-	9.863	10.290
SLS-SOO/54:2	-	-	1.849	1.877
SOO/54:2	6.570	2.317	-	-
SOP/52:1	5.177	-	-	-

Note: L = linoleic acid; M = myristic acid; O = oleic acid; P = palmitic acid; S = stearic acid; - = values below the detection limit.

**Table 3:** Quality parameters of açaí, guava, passion fruit, and pequi oils.

Property	Açaí	Guava	Passion Fruit	Pequi
Acidity index (mg KOH/g)	6.17 ± 0.03	2.12 ± 0.02	0.45 ± 0.01	5.25 ± 0.13
Peroxide index (meq/kg)	9.778 ± 0.005	11.567 ± 0.029	7.905 ± 0.002	9.600 ± 0.005
Saponification index (mg KOH/g)	184.50	191.01	181.26	184.54
Iodine index	114.13	134.25	121.76	110.02
Refractive index	1.463 ± 0.045	1.465 ± 0.005	1.464 ± 0.004	1.464 ± 0.045
Oxidative stability index (h) at 110 °C	15.24 ± 0.01	16.04 ± 0.01	10.05 ± 0.04	15.49 ± 0.02
Kinematic viscosity (mm^2^/s) *	43.0 ± 0.1	35.9 ± 0.1	32.8 ± 0.01	36.8 ± 0.02

Note: Values are expressed as mean ± SD of three replicates. * Measured at 40 °C.

**Table 4:** Main results of thermogravimetric analysis of pequi, açaí, guava, and passion fruit oils in synthetic air and nitrogen atmospheres. T = temperature.

Synthetic Air Atmosphere									Nitrogen Atmosphere		
	1st Step		2nd Step		3rd Step		4th Step			1st Step	
Oil	T range (°C)	Mass loss (%)	T range (°C)	Mass loss (%)	T range (°C)	Mass loss (%)	T range (°C)	Mass loss (%)	Oil	T range (°C)	Mass loss (%)
Guava	222.33–364.47	10.93	326.76–373.63	44.141	424.88–44385	27.775	528.96–563.5	0.518	Guava	311.8–495.83	96.75
Passion fruit	218.99–269.73	8.745	330.59–387.59	45.729	420.30–452.19	28.9	531.29–580.39	12.373	Passion fruit	281.15–481	91.256
Pequi	298.30–379.37	59.696	417.33–453.99	24.2	212.30–566.71	13.47	-	-	Pequi	343.6–482.85	99.33
Açaí	382.60–443.41	96.309	-	-	-	-	-	-	Açaí	332.32–468.11	99.45

## Data Availability

The original contributions presented in the study are included in the article, further inquiries can be directed to the corresponding author.
